# 609 Available non-invasive skin probes distinguish between normal skin and hypertrophic scar but not laser-treated scar

**DOI:** 10.1093/jbcr/irac012.237

**Published:** 2022-03-23

**Authors:** Bonnie C Carney, Lesle M Jimenez, Mary A Oliver, John W Keyloun, Lauren T Moffatt, Jeffrey W Shupp, Taryn E Travis

**Affiliations:** Medstar Health Research Institute, Washington DC, District of Columbia; MedStar Health Research Institute, Washington, District of Columbia; Burn Center at MedStar Washington Hospital Center, Washington DC, District of Columbia; Burn Center at MedStar Washington Hospital Center, Washington DC, District of Columbia; Burn Center at MedStar Washington Hospital Center, Washington DC, District of Columbia; MedStar Washington Hospital Center, Washington DC, District of Columbia; MedStar Washington Hospital Center, Washington, District of Columbia

## Abstract

**Introduction:**

Skin fibrosis is the most under-studied fibrotic condition, and as such, there are limited treatment options for hypertrophic scar (HTS). HTS is difficult to study because scars improve over time, thus making the distinction between natural improvements and interventions difficult. In addition, clinical, histologic, cellular, and molecular outcome metrics are not agreed upon among providers/researchers. It was hypothesized that a set of non-invasive skin probes would be able to distinguish between HTS and normal skin (NS), but not between HTS before and after treatment with fractional ablative laser, despite histologic-level evidence showing improvement with treatment.

**Methods:**

Wounds were created and HTS were allowed to form (n=8 scars). At Day 77, HTS and NS (n=4) were assessed with non-invasive skin probes measuring elasticity and trans-epidermal water loss (TEWL). HTS were then treated with CO_2_ fractional ablative laser (FLSR) at 70 mJ, 250Hz, and 1% density. The same data, sample collection, and FLSR treatment was carried out at weeks 1, 2, 3 and 4. Formalin-fixed biopsies were processed and stained with H&E, Herovici, Masson’s Trichrome, and Picrosirius red stains. Collagen type and architecture were qualitatively evaluated. Rete ridge ratio (RRR) was calculated from H&E stains.

**Results:**

All stains distinguished between HTS and NS with ease. Herovici stain showed differences in collagen architecture and collagen type after treatment with FLSR. Pre laser treatment, elasticity was different between HTS and NS (290.50±32.90 N/m vs. 90.92±5.61 N/m, p< 0.001). However, elasticity did not change after FLSR at 1, 2, 3, or 4 weeks (248.17±48.54, 215.54±44.68, 295.29±37.5, 290.79±30.17, p >0.05). RRR was different between HTS and NS (1.22±0.11 vs. 1.51±0.03, p=0.08). FLSR induced a significant increase in RRR in HTSs (pre=1.3±0.1 vs. week 4=1.9±0.1, p< 0.05). Pre-treatment, TEWL was different between HTS and NS (12.96±1.99 g/m^2^h vs. 8.72±1.11 g/m^2^h, p=0.09). However, TEWL did not change after FLSR at 1, 2, 3, or 4 weeks (15.95±1.99, 20.35± 3.15, 15.9±1.96, 14.01±2.22, p >0.05).

**Conclusions:**

Outcome metrics that non-invasively measure the qualities of scar are critical for HTS research to evaluate treatment effectiveness. Currently available technologies distinguish between NS and HTS well. However, they are inadequate to distinguish scars pre- and post-treatment despite evidence at the histologic level that changes occur. New technologies should be developed that are able to more effectively demonstrate changes to HTS after treatment.

